# Exploring Milk and Blood Biochemical Indicators as Potential Biomarkers of Udder Health in Early Lactation Cows

**DOI:** 10.3390/vetsci12121138

**Published:** 2025-11-29

**Authors:** Akvilė Girdauskaitė, Samanta Grigė, Eimantas Ginkus, Karina Džermeikaitė, Justina Krištolaitytė, Ieva Rodaitė, Greta Šertvytytė, Lina Anskienė, Gabija Lembovičiūtė, Ramūnas Antanaitis

**Affiliations:** 1 Large Animal Clinic, Veterinary Faculty, Lithuanian University of Health Sciences, Tilzes Str. 18, LT-47181 Kaunas, Lithuania; samanta.grige1@lsmu.lt (S.G.); eimantas.ginkus@lsmu.lt (E.G.); gabija.lemboviciute@lsmu.lt (G.L.);; 2 Department of Animal Breeding, Faculty of Animal Sciences, Lithuanian University of Health Sciences, Tilzes Str. 18, LT-47181 Kaunas, Lithuania

**Keywords:** somatic cell count, dairy cows, automated monitoring, innovative technologies, automatic milking system (AMS)

## Abstract

SCC is a standard indicator of udder inflammation, but it reflects only part of the broader physiological changes occurring in the mammary gland. This study aimed to evaluate associations between SCC, in-line milk traits, and blood biochemical markers in Holstein dairy cows. Based on SCC and California Mastitis Test (CMT) results, 59 cows (20–100 DIM) were divided into three groups: Group 1 (SCC < 200,000 cells/mL; *n* = 20), Group 2 (SCC 200,000–500,000 cells/mL; *n* = 19), and Group 3 (SCC > 500,000 cells/mL; *n* = 20). The Lely Astronaut^®^ A3 system was used to record milk parameters and behavioral data, while blood samples were collected for biochemical analysis. While there were negative relationships with milk yield (r = −0.266, *p* < 0.05) and creatinine (r = −0.291, *p* < 0.05), there was a significant positive correlation between SCC and milk electrical conductivity (EC) (r = 0.330, *p* < 0.05), gamma-glutamyl transferase (GGT) (r = 0.424, *p* < 0.001), and lactate dehydrogenase (LDH) (r = 0.285, *p* < 0.05). Potassium and chloride concentrations varied between groups, indicating slight electrolyte imbalances linked to higher SCC even though they remained within physiological bounds. Receiver operating characteristic (ROC) analysis further showed that milk EC (area under the curve (AUC) = 0.770) and blood potassium (AUC = 0.707) demonstrated the highest diagnostic accuracy for distinguishing healthy and mastitic cows. These results show that integrating SCC data with automated in-line monitoring and blood biochemical profiling can help identify novel complementary indicators for the detection of mastitis in dairy cows and offer a deeper understanding of udder health.

## Simple Summary

Mastitis is a widespread problem in dairy herds, and its early detection remains a challenge, especially when the disease develops without clear clinical signs. While somatic cell count (SCC) is routinely employed to assess udder health, growing attention is being given to other milk and blood indicators that could help identify inflammation at an earlier stage. In this study, we evaluated whether combining automated in-line milk measurements with blood biochemical profiling could reveal additional markers linked to udder health. We examined 59 early-lactation Holstein cows (20–100 days in milk (DIM)) and compared their milk parameters and blood test results across different SCC levels. Cows with higher SCC showed increased milk electrical conductivity and small but consistent shifts in blood electrolytes and metabolites, particularly potassium and creatinine. Although none of these indicators worked as a stand-alone diagnostic tool, their combined evaluation provided a clearer picture of the physiological changes occurring during udder inflammation. These findings support ongoing efforts to identify new complementary biomarkers that could improve the early detection and monitoring of mastitis in dairy cows.

## 1. Introduction

Mastitis remains one of the most frequent health problems in dairy cattle and continues to receive attention because many cases develop without clear clinical signs, yet still affect milk quality and cow performance. Although SCC is the most widely used indicator of intramammary inflammation, it is increasingly recognized that SCC alone does not fully reflect the range of physiological changes taking place in the mammary gland during disease [1]. Recent reviews emphasize the need to broaden mastitis diagnostics and to consider additional milk- and blood-derived traits that may complement SCC and help identify inflammation at an earlier stage [2].

Continuous, uniform in-line monitoring of physiological markers and milk composition is made possible by automatic milking systems. Several of these routinely measured traits have been investigated as potential markers of udder health [3]. Electrical conductivity is one of the most consistently associated indicators, as changes in ion balance occur early in the inflammatory process and often precede visible alterations in milk [4]. Lactose concentration has also gained attention: lower lactose levels are repeatedly reported in cows with subclinical mastitis and may indicate impaired epithelial integrity [5,6]. Other routinely recorded traits, such as milk temperature, fat-to-protein ratio or daily milk yield, likewise reflect physiological responses that may accompany inflammation or metabolic stress, particularly in early lactation [7]. More recently, multivariate approaches combining several milk indicators have been proposed as a more informative way to describe udder health, especially when different traits are interpreted together rather than in isolation [8]. Metabolomic studies have further expanded mastitis biomarker research by identifying specific milk metabolites that differ between healthy and mastitic cows, supporting the idea that milk contains numerous potential biomarkers beyond SCC [9,10].

Changes associated with udder inflammation are not limited to milk composition. Blood biochemical indicators may reflect systemic responses to mastitis and therefore offer an additional perspective [11]. Enzymes such as gamma-glutamyl transferase, lactate dehydrogenase and alanine phosphatase have been reported to increase during inflammatory conditions, suggesting links between mammary health and wider metabolic or hepatic responses [12,13]. Electrolytes, including potassium and chloride, may also vary in cows experiencing inflammatory or metabolic challenges. Recent work has documented that some of these indicators show parallel changes in milk and blood, pointing to a coordinated physiological reaction rather than an isolated local process [14]. Despite these findings, the relationships between blood biochemistry, routinely recorded milk traits and SCC are still not well described in early-lactation cows.

As automated milk monitoring systems become more widely available and blood biochemical profiling is routinely used in dairy practice, there is growing interest in integrating these data sources. This combined approach may offer a broader perspective on udder health. In this context, the aim of the present study was to investigate the associations between SCC, in-line milk parameters and blood biochemical traits, and to assess the potential of these indicators as adjunctive markers of udder inflammation in early-lactation dairy cows.

## 2. Materials and Methods

### 2.1. Study Animal Housing Conditions

The study adhered to the Lithuanian Law on Animal Welfare and Protection and was approved under the number G2-298. The study was conducted on a commercial dairy farm in the Lithuanian region of Kaunas, and the Lithuanian University of Health Sciences’ Large Animal Clinic processed the data. The experiment ran from 1 September 2025 to 15 September 2025. For this study, 59 Holstein dairy cows (41 multiparous and 18 primiparous) were chosen for close observation. With a mean of 58 DIM, the cows’ DIM ranged from 20 to 100. The cows were 2 to 6 years old, with an average age of 4.1 years. The average daily milk yield of the cows was approximately 35 kg per day. Cows were selected to represent both healthy and mastitic animals; those with systemic diseases or under treatment were excluded. To verify their health status and make sure there were no systemic diseases or other incapacitating conditions, the same veterinarian examined each cow in accordance with a standardized clinical protocol. As a result, cows with subclinical or clinical mastitis or those in clinical health were included in the study. There were approximately 1300 lactating Holstein cows on the farm, which were milked using 20 Lely Astronaut^®^ A3 milking robots (Lely, Maassluis, The Netherlands). The cows weighed between 550 and 650 kg on average. They were housed in free-stall barns with ventilation. The average yearly milk production in 2024 was 11,904 kg per cow, with a mean protein content of 3.6% and a fat content of 4.2%. All year long, the cows were kept in a loose system and fed a total mixed ration (TMR) that was tailored to meet their physiological needs. Feeding took place twice a day, at 7:00 and 15:00. The milking robots gave the cows 2 kg of concentrate feed every day in addition to their base ration, and an automated feed pusher redistributed the feed 22 times a day (Lely Juno, Lely, Maassluis, The Netherlands). Clean drinking water was available to cows at all times. Throughout the study period, all cows received the same TMR, composed of corn silage, grass hay, grass silage, grain concentrate mash, and a mineral mix (Table 1 and Table 2). This high proportion of grain concentrate mash (50%) is normal for this farm and is routinely used to support the nutritional needs of high-yielding cows.

### 2.2. Grouping of Animals

According to established literature and based on SCC and California Mastitis Test (CMT) results (negative, trace, +, ++, +++), 59 cows were categorized into three groups: Group 1 (SCC < 200,000 cells/mL; *n* = 20; 14 multiparous, 6 primiparous) was considered healthy because they did not exhibit any clinical signs of mastitis and tested negative on the CMT; Group 2 (SCC 200,000–500,000 cells/mL; *n* = 19; 13 multiparous, 6 primiparous) had a positive CMT reaction but no outward signs of mastitis; and Group 3 (SCC > 500,000 cells/mL; *n* = 20; 14 multiparous, 6 primiparous) had positive CMT results and visible clinical changes in the milk. These SCC thresholds follow commonly used classification criteria in mastitis research and are consistent with the grouping described by Guan et al. [15]. Data collection was preceded by the grouping process.

### 2.3. Parameter Registration

This study used the Lely Astronaut^®^ A3 milking system (Lely, Maassluis, The Netherlands) to measure lactose, milk temperature, electrical conductivity, fat, protein, fat-to-protein ratio, and rumination time. Each cow was assigned to one of the three SCC-based groups at the same time that clinical examinations and coccygeal vein blood sampling were completed on the same day by a licensed veterinarian. Therefore, on the same day, each animal’s blood biochemical indicators, milk parameters, and clinical evaluation results were gathered. Because the cows were milked in the automatic milking system, milk parameters represented composite samples from all four udder quarters. The system integrates all milkings performed during 24 h, providing a daily average value rather than measurements from a single morning or evening milking.

### 2.4. Collected Variables

During every milking session, the Lely Astronaut^®^ A3 milking system (Lely, Maassluis, The Netherlands) automatically recorded a number of milk parameters. In-line sensors built into the milking claw were used to measure milk temperature (°C) and electrical conductivity (mS/cm). The system’s near-infrared optical sensor measured the contents of lactose (%), fat (%), and protein (%), from which the fat-to-protein ratio was computed. The system’s neck sensor was used to record the amount of time spent ruminating, which was then expressed as minutes per 24 h (min/24 h). All milk composition parameters were obtained directly from the in-line sensors of the Lely Astronaut^®^ A3 system, and no additional laboratory chemical analyses were performed. Additionally, the system automatically recorded the rumination time (min/24 h). Every cow had one measurement of each parameter on the day of the clinical examination and the animal’s assignment to one of the three SCC-based groups.

On the day of grouping, blood samples were taken from each cow’s coccygeal vein during the clinical examination. BD Vacutainer^®^ serum tubes (Becton Dickinson, Eysins, Switzerland) without anticoagulant were used for blood sample collection. The samples were taken to the Lithuanian University of Health Sciences’ Veterinary Academy’s Large Animal Clinic’s Laboratory of Clinical Tests. After centrifugation at 1500× *g* for 15 min, the serum samples were analyzed in the laboratory using a fully automated wet-chemistry analyzer, the RX Daytona™ (Randox Laboratories Ltd., London, UK), equipped with dedicated Randox biochemical reagent kits. The biochemical profile included measurements of albumin (ALB), alanine aminotransferase (ALT), aspartate aminotransferase (AST), calcium (Ca), creatinine (CREA), C-reactive protein (CRP), iron (Fe), GGT, glucose (GLUC), LDH, magnesium (Mg), non-esterified fatty acids (NEFA), phosphorus (PHOS), total protein (TP), triglycerides (TRIG), and urea (UREA), as well as electrolytes: sodium (Na), potassium (K), and chloride (Cl).

### 2.5. Statistical Analysis

Statistical analyses were performed using SPSS, IBM SPSS Statistics 30 (SPSS Inc., Chicago, IL, USA). For analysis, SCC data were log-transformed to SCClog10 to normalize the distribution of the variable, as somatic cell count data are typically right-skewed [16]. Descriptive statistics are presented as means ± standard error of the mean (SEM). Comparisons of means among SCC groups were conducted using one-way analysis of variance (ANOVA), followed by Tukey’s post hoc test for multiple comparisons. The relationships between SCC and the investigated milk and blood indicators were assessed using Pearson correlation analysis. To further investigate potential systemic indicators of udder health, a binary logistic regression model was constructed with SCC group low (Group 1) vs. high (Group 3) SCC as the dependent variable. Statistical significance was set at *p* < 0.05. Because the present study was exploratory and based on a limited sample size, effect size measures (e.g., Cohen’s d) were not calculated.

To evaluate the diagnostic performance of milk parameters in distinguishing cows with healthy udders from those with severe mastitis, an ROC analysis was performed. Initially, cows were categorized into three groups based on somatic cell count (SCC): Group 1 (SCC < 200,000 cells/mL; *n* = 20), Group 2 (SCC 200,000–500,000 cells/mL; *n* = 19), and Group 3 (SCC > 500,000 cells/mL; *n* = 20). For ROC analysis, only cows from Group 1 and Group 3 were included to create a binary classification (healthy vs. mastitic), as the ROC method requires two distinct states. Group 1 was coded as 0 (negative state), and Group 3 as 1 (positive state).

The diagnostic accuracy of individual milk and blood markers in differentiating healthy cows from those with mastitis was assessed using receiver operating characteristic (ROC) analysis. By plotting sensitivity against 1-specificity across all potential cut-off values, ROC curves enable evaluation of a parameter’s ability to distinguish between two health states. This performance is measured by the area under the ROC curve (AUC): an AUC of 0.5 denotes no discriminatory capacity, 0.6–0.7 poor, 0.7–0.8 acceptable, 0.8–0.9 very good, and >0.9 excellent discrimination. The Youden index (sensitivity + specificity − 1), which establishes the cut-off that offers the best balance between sensitivity and specificity, was used to find the ideal threshold for each parameter. For each ROC curve, the 95% confidence intervals (CI) for the AUC were calculated.

## 3. Results

### 3.1. Automated Monitoring Parameters (Mean ± SEM) in Cows Grouped by SCC

Statistically significant differences were observed in electrical conductivity of milk. Electrical conductivity was significantly higher in cows from Group 3 compared to Group 1, representing a +5.9% increase (*p* < 0.01). No other indicators showed statistically significant differences between groups *p* > 0.05 (Table 3).

### 3.2. Blood Biochemical Indicators (Mean ± SEM) in Cows Grouped by SCC

Creatinine levels in Group 3 were 12.5% lower than in Group 1 (*p* < 0.05). Potassium levels were 7.5% higher in Group 2 compared to Group 1 (*p* < 0.01), and 6.3% higher in Group 3 compared to Group 1 (*p* < 0.05). Chloride levels were 5.6% higher in Group 2 compared to Group 1 (*p* < 0.05). All other indicators showed percentage changes between groups ranging from ±1% to ±30%, but these differences were not statistically significant (Table 4).

### 3.3. Correlation of SCC with Milk Parameters

**Figure 1:**
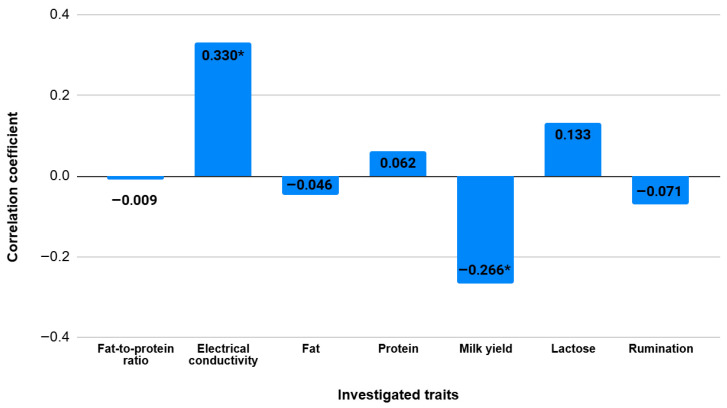
Correlation coefficients between SCC and milk parameters (* *p* < 0.05).

The associations between automated milk parameters and SCC were assessed using a thorough correlation analysis (Figure 1). There were both positive and negative associations indicated by the correlation coefficients, which varied from −0.266 to 0.330. Milk electrical conductivity showed a significant positive correlation with SCC (r = 0.330, *p* < 0.05), whereas milk yield demonstrated a clear negative correlation with SCC (r = −0.266, *p* < 0.05). SCC did not exhibit statistically significant correlations with any other milk-related parameters.

### 3.4. Correlation of SCC with Blood Parameters

**Figure 2:**
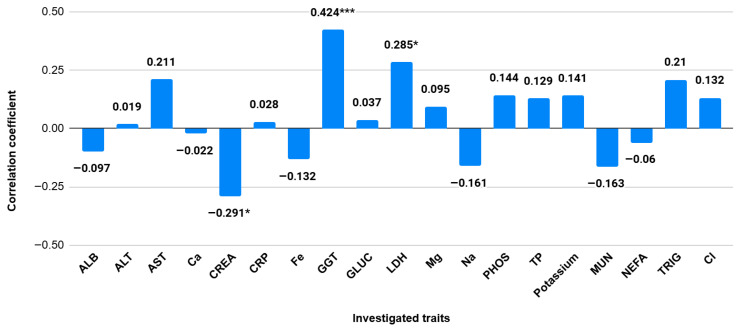
Correlation coefficients between SCC and blood parameters (*** *p* < 0.01, * *p* < 0.05).

Correlation analysis revealed several notable associations between somatic cell count and blood biochemical indicators (Figure 2). The strongest blood-based association was observed between SCC and GGT (r = 0.424, *p* < 0.001), while LDH also showed a weaker positive correlation with SCC (r = 0.285, *p* < 0.05). In contrast, creatinine demonstrated a moderate negative correlation with SCC (r = −0.291, *p* < 0.05).

### 3.5. Diagnostic Evaluation of Milk and Blood Biomarkers Using ROC Analysis

Among the investigated milk traits, electrical conductivity of milk demonstrated the highest diagnostic accuracy (AUC = 0.770; 95% CI: 0.619–0.921; *p* < 0.001), indicating good discriminatory ability between healthy and mastitic cows. All other parameters showed AUC values close to 0.5, suggesting poor or no discrimination. Specifically, fat-to-protein ratio (AUC = 0.548), fat percentage (AUC = 0.519), protein percentage (AUC = 0.474), and rumination time (AUC = 0.501) exhibited weak performance. Milk yield (AUC = 0.398), lactose (AUC = 0.351), and milk temperature (AUC = 0.359) had the lowest AUC values, indicating very poor diagnostic potential (Table 5).

ROC analysis was performed to assess the ability of blood biochemical parameters to discriminate between cows with low SCC (Group 1) (<200,000 cells/mL) and high SCC (Group 3) (>500,000 cells/mL). Among the tested biomarkers, potassium exhibited the highest diagnostic accuracy (AUC = 0.707; 95% CI: 0.542–0.871; *p* < 0.05), indicating good discrimination between healthy and mastitic cows. Magnesium (AUC = 0.662; 95% CI: 0.488–0.835; *p* = 0.067), chloride (AUC = 0.662; 95% CI: 0.484–0.839; *p* = 0.074), and total protein (TP) (AUC = 0.657; 95% CI: 0.482–0.831; *p* = 0.079) The remaining investigated parameters had AUC values close to 0.5, indicating poor or no discriminatory ability (Table 6).

## 4. Discussion

In order to find possible biomarkers for mastitis diagnosis, the current study sought to examine the connections between SCC, in-line milk monitoring data, and blood biochemical indicators. In this context, our results highlight which milk- and blood-derived indicators may serve as potential biomarkers of udder inflammation in early-lactation cows. It also assessed variations in these parameters among three SCC-based groups of cows. In our study, a significant positive correlation was found between SCC and milk electrical conductivity (r = 0.330, *p* < 0.01), and cows in group 3 showed on average 5.9% higher conductivity values compared with cows in group 1. Inflammatory responses increase cellular breakdown products that modify the ionic composition of milk, thereby raising electrical conductivity [17,18]. Consistent with these mechanisms, previous studies have shown that EC increases in parallel with SCC, particularly in subclinical mastitis cases. Among all milk traits, EC showed the highest AUC (0.770), confirming its strong association with SCC-based group differences and supporting previous evidence that ionic shifts make EC a sensitive mastitis indicator [3,19]. In contrast, other milk parameters, such as the fat-to-protein ratio and lactose, had AUC values close to 0.5, indicating limited discriminatory value.

Another parameter recorded by the automated milking system in our study was milk yield, which showed a significant negative correlation with somatic cell count (r = −0.266, *p* < 0.05). Previous studies likewise report a clear negative association between milk yield and SCC, supporting our findings that higher SCC is linked to reduced production [20,21]. According to Kul et al. [22], elevated SCC adversely affected milk component concentrations, indicating an antagonistic relationship between milk yield and milk quality as SCC grows, even though milk yield may initially increase during early lactation. For example, Johnson and Young [23] found a negative correlation between SCC and milk production-related parameters, supporting the idea that higher SCC is associated with lower milk yield and quality. In dairy operations, this pattern of lower yield with SCC is essential for controlling and enhancing the general health and productivity of the herd.

Cows in Group 1 had 12.5% higher quantities of creatinine than those in Group 3, indicating a negative correlation between creatinine and SCC in our study (r = −0.291, *p* < 0.05). This is consistent with previous findings that metabolic waste products, such as creatinine, might be impacted by increased SCC and intramammary inflammation [24,25]. Reduced creatinine concentrations in mastitis or stressed cows have also been linked to changes in metabolic pathways and inflammatory activation [26,27]. According to some scientists, cows with inflamed udders may also show decreased physiological resilience or metabolic efficiency, which could further drop creatinine levels [28,29]. Larger and longer-term datasets are still needed to establish the link, even if these mechanisms offer a tenable explanation for the pattern seen in our analysis.

In our study, a significant positive correlation was observed between SCC and GGT activity (r = 0.424, *p* < 0.001), indicating that cows with higher SCC values had increased GGT levels. GGT concentration frequently rises in cows with elevated SCC, indicating a greater metabolic stress response brought on by the inflammatory state brought on by an infection [30]. Previous work also shows that GGT increases under physiological stress, which aligns with the idea that elevated SCC reflects underlying intramammary inflammation affecting metabolic enzyme activity [31]. Examining the underlying mechanisms of inflammation. Elevated GGT levels can reflect the severity of the metabolic disturbances associated with mastitis. [32]. The significance of combining blood-derived indicators with milk parameters is supported by similar patterns that have been reported, demonstrating that systemic biochemical changes are linked to differences in milk production and composition attributes [33].

In our study, SCC was also significantly positively correlated with LDH activity (r = 0.285, *p* < 0.05), showing that cows with higher SCC levels had elevated LDH concentrations. Inflammation and tissue injury in the udder are reflected in LDH activity [34]. Similarly, Antanaitis et al. [31] found a substantial correlation between LDH in milk and SCC, indicating that it can be used as a diagnostic tool for mastitis in the management of dairy herds. Monitoring both SCC and LDH may facilitate earlier detection and treatment of mastitis, improving cow health and overall production efficiency. Of the blood biomarkers analyzed, potassium showed the highest AUC value (0.707) among blood indicators, suggesting moderate association with SCC-based groups. GGT exhibited poor diagnostic performance (AUC = 0.564), while urea (AUC = 0.447), AST (AUC = 0.487), and NEFA (AUC = 0.526) demonstrated no meaningful discriminatory capacity. These results are consistent with previous studies highlighting electrolyte imbalance and liver enzyme activity as systemic indicators of mastitis [35]. Together, these findings suggest that, although electrolyte alterations—particularly in potassium concentration—may reflect inflammatory processes associated with mastitis, most blood biochemical indicators lack sufficient diagnostic precision to be used independently. Therefore, in-line measures should not be used in place of comprehensive laboratory milk analysis because they cannot fully characterize somatic cell composition or detect specific inflammatory mediators.

In our study, all measured potassium and chloride concentrations were within the physiological range; however, significant group differences were observed. These electrolyte variations may reflect the broader metabolic strain characteristic of high-yielding cows in early lactation, when increased nutrient demand and systemic inflammatory responses can influence mineral balance and hepatic enzyme activity [28,29]. Potassium levels were 7.5% higher in group 2 (*p* < 0.01) and 6.3% higher in group 3 (*p* < 0.05) compared with group 1, while chloride concentrations were 5.6% higher in group 2 than in group 1 (*p* < 0.05). These findings suggest that elevated SCC may be linked to slight alterations in electrolyte balance. Some research has documented electrolyte imbalances in mastitic cows, including raised serum chloride levels, whereas other studies have demonstrated that biochemical changes in cows with elevated SCC may be due to inflammatory stress influencing electrolyte homeostasis [36]. Thus, the observed variations in potassium and chloride concentrations might be indicative of metabolic and inflammatory alterations linked to elevated SCC, albeit further focused research is required to validate these connections. These variations suggest that some blood electrolytes may participate in systemic inflammatory responses associated with udder health. When combined, these results show that a number of the measured indicators—specifically, EC, LDH, GGT, and potassium—are associated with inflammatory processes in the udder and may provide supportive information for future mastitis monitoring approaches.

The majority of milk and blood associations with SCC were poor, indicating the complexity of physiological reactions to udder inflammation, despite differences in creatinine, potassium, and electrical conductivity between SCC groups. However, integrating in-line sensor characteristics with blood biochemical markers offers a more comprehensive understanding of how inflammatory processes impact cow physiology and may facilitate more insightful health-monitoring strategies. The ROC results also imply that a number of characteristics, especially potassium and electrical conductivity, may help identify cows with severe udder inflammation. To confirm these correlations and assess their applicability for AMS-based and Precision Livestock Farming (PLF) oriented monitoring systems, larger, longer-term studies are needed.

The possible application of these results in PLF systems in the future is a practical implication. A practical translation pathway would involve incorporating these biochemical indicators into existing AMS sensor streams, enabling their integration into farm-level dashboards where deviations could trigger automated alerts or inform decision-support tools. In the future, real-time monitoring methods may include several of the biochemical markers linked to increased SCC in this study, namely GGT, LDH, potassium, and creatinine. The ability of contemporary AMS units and wearable technology to continuously gather physiological and behavioral data is growing, and incorporating these markers would facilitate the early detection of cows under metabolic or inflammatory stress [36]. These systems may be strengthened further by combining biochemical data with behavioral or time-activity data acquired using sophisticated AI-based analytical techniques. This perspective is supported by recent work demonstrating that AI models can reliably extract detailed behavioral patterns from PLF sensor outputs, offering a feasible pathway for more comprehensive, data-driven health surveillance on farms [37,38].

The relatively small sample size and the unequal distribution of cows among the three SCC groups limit the statistical power of the current investigation and weaken the validity of between-group comparisons. Since all measurements were taken at a single time point during early lactation, it is not possible to examine temporal changes or the markers’ capacity to forecast future udder health outcomes, hence the data should be viewed as exploratory rather than predictive. Additionally, despite the fact that different bacterial infections may cause varied biochemical and inflammatory responses, cows were not categorized based on the causal pathogen; this may have contributed to the dataset’s heterogeneity. Future research should use larger cohorts, longitudinal sampling, and pathogen-specific diagnostics to increase the generalizability and reproducibility of these correlations.

## 5. Conclusions

This study shows that adding blood biochemical profile to routinely collected in-line milk parameters provides more information on physiological changes related to udder health in early-lactation cows. A number of indicators, such as milk electrical conductivity and serum potassium, consistently varied between cows with low and high somatic cell counts, even though the associations were generally weak. This suggests that these indicators may represent more general inflammatory or metabolic reactions that take place during mastitis.

The observed findings should not be taken as diagnostic criteria but rather as early evidence that numerous physiological systems respond concurrently to udder inflammation. This supports the potential for creating more informative monitoring frameworks in automated milking systems by integrating milk sensor outputs with specific blood indicators. However, the restricted sample size and single-time-point design limit how broadly these findings may be applied. Future research using longitudinal designs, bigger cow populations, and pathogen-specific characterization will be necessary to assess whether these combined indicators might lead to more precise and timely detection of udder health issues in dairy herds.

## Figures and Tables

**Table 1:** Composition of the TMR fed to the study cows.

TMR Component	Value
Corn silage	30%
Grass hay	4%
Grass silage	10%
Grain concentrate mash	50%
Mineral mix	6%

**Table 2:** Chemical composition of TMR.

TMR Component	Value
Dry Matter (DM)	47.8%
Neutral Detergent Fiber (NDF)	29% of DM
Acid Detergent Fiber (ADF)	17.5% of DM
Non-Fiber Carbohydrates (NFC)	38.6% of DM
Crude Protein (CP)	15.8% of DM

**Table 3:** Mean ± SEM of automated monitoring parameters across SCC groups (** *p* < 0.01).

Investigated Trait	Group 1	Group 2	Group 3
Electrical conductivity of milk (mS/cm)	68.65 ± 0.737 **	70.21 ± 0.897	72.70 ± 1.029 **
Fat-to-protein ratio	1.09 ± 0.059	1.18 ± 0.053	1.13 ± 0.048
Fat (%)	3.77 ± 0.195	4.03 ± 0.154	3.83 ± 0.174
Protein (%)	3.48 ± 0.076	3.43 ± 0.053	3.69 ± 0.308
Milk yield (kg/day)	43.89 ± 3.345	40.26 ± 2.834	38.61 ± 2.540
Lactose (%)	4.59 ± 0.021	4.56 ± 0.022	4.56 ± 0.054
Rumination time (min/24 h)	515.30 ± 22.739	543.58 ± 19.303	514.15 ± 25.489

**Table 4:** Mean ± SEM of blood biochemical indicators across SCC groups (** *p* < 0.01, * *p* < 0.05).

Investigated Trait	Units	Standard	Group 1	Group 2	Group 3
Albumin (ALB)	g/L	30–40	33.471 ± 1.261	34.168 ± 0.673	32.197 ± 0.988
Alanine Phosphatase (ALT)	U/L	0–50	26.983 ± 2.458	28.882 ± 1.672	26.734 ± 1.502
Aspartate Aminotransferase (AST)	U/L	0–80	89.124 ± 9.592	81.882 ± 4.554	93.036 ± 9.783
Calcium (Ca)	mmol/L	2.2–2.9	2.245 ± 0.056	2.311 ± 0.033	2.239 ± 0.041
Creatinine (CREA)	µmol/L	88.4–176.8	54.187 ± 2.231 *	52.503 ± 1.531	47.398 ± 1.509 *
C-Reactive Protein (CRP)	mg/L	<10	12.204 ± 1.047	14.274 ± 1.010	12.605 ± 0.761
Iron (Fe)	µmol/L	27–40	22.184 ± 1.148	19.474 ± 1.022	19.452 ± 1.502
Gamma-Glutamyl Transferase (GGT)	U/L	0–20	31.552 ± 3.045	31.532 ± 1.809	41.243 ± 7.304
Glucose (GLUC)	mmol/L	2.5–3.3	2.731 ± 0.095	3.047 ± 0.077	2.951 ± 0.116
Lactate Dehydrogenase (LDH)	U/L	<1500	1443.429 ± 133.989	1217.478 ± 58.993	1288.404 ± 90.783
Magnesium (Mg)	mmol/L	0.8–1.2	0.967 ± 0.037	1.039 ± 0.038	1.042 ± 0.032
Non-Esterified Fatty Acids (NEFA)	mmol/L	0.1–0.79	0.049 ± 0.012	0.045 ± 0.013	0.037 ± 0.007
Phosphorus (PHOS)	mmol/L	1.6–2.3	1.946 ± 0.106	2.005 ± 0.072	2.018 ± 0.071
Total protein (TP)	g/L	50–80	70.675 ± 2.075	76.386 ± 1.651	75.258 ± 2.128
Triglycerides (TRIG)	mmol/L	0.17–0.51	0.128 ± 0.007	0.129 ± 0.007	0.126 ± 0.005
Milk Urea Nitrogen (MUN)	mmol/L	3.3–5	5.311 ± 0.235	5.468 ± 0.159	5.113 ± 0.251
Natrium (Na)	mmol/L	135–145	132.264 ± 2.066	137.952 ± 1.906	129.502 ± 6.363
Potassium	mmol/L	3.5–4.5	4.152 ± 0.082 **	4.466 ± 0.076 *	4.411 ± 0.067 **
Chloride (Cl)	mmol/L	90–110	90.329 ± 1.658 *	95.371 ± 1.307 *	94.051 ± 1.239

**Table 5:** Area under the ROC curve (AUC) for milk parameters used to discriminate between low (Group 1) and high (Group 3) SCC groups (*** *p* < 0.001).

Milk Parameters	AUC	Std. Error	Asymptotic Sig.	Asymptotic 95% Confidence Interval	
				Lower Bound	Upper Bound
Electrical conductivity of milk (mS/cm)	0.770 ***	0.077	0.000	0.619	0.921
Fat-to-protein ratio	0.548	0.094	0.612	0.364	0.731
Fat (%)	0.519	0.093	0.841	0.336	0.702
Protein (%)	0.474	0.094	0.780	0.289	0.658
Milk yield (kg/day)	0.398	0.098	0.295	0.206	0.589
Lactose (%)	0.351	0.089	0.094	0.177	0.525
Rumination time (min/24 h)	0.501	0.093	0.989	0.319	0.684
Milk temperature (°C)	0.359	0.088	0.109	0.186	0.532

**Table 6:** Area under the ROC curve (AUC) for blood parameters used to discriminate between low (Group 1) and high (Group 3) SCC groups (* *p* < 0.05).

Blood Parameters	Units	AUC	Std. Error	Asymptotic Sig.	Asymptotic 95% Confidence Interval	
					Lower Bound	Upper Bound
ALB	g/L	0.407	0.093	0.318	0.223	0.590
ALT	U/L	0.543	0.095	0.647	0.358	0.729
AST	U/L	0.487	0.096	0.891	0.299	0.675
Ca	mmol/L	0.468	0.094	0.738	0.283	0.654
CREA	umol/L	0.287	0.086	0.013	0.119	0.454
CRP	mg/L	0.516	0.096	0.869	0.328	0.703
Fe	umol/L	0.379	0.092	0.190	0.198	0.560
GGT	U/L	0.564	0.094	0.491	0.381	0.748
GLUC	mmol/L	0.609	0.091	0.230	0.431	0.788
LDH	U/L	0.439	0.094	0.519	0.256	0.623
Mg	mmol/L	0.662	0.089	0.067	0.488	0.835
NEFA	mmol/L	0.526	0.094	0.780	0.341	0.711
PHOS	mmol/L	0.516	0.095	0.869	0.329	0.703
TP	g/L	0.657	0.089	0.079	0.482	0.831
TRIG	mmol/L	0.607	0.095	0.263	0.420	0.793
Milk Urea Nitrogen (MUN)	mmol/L	0.447	0.094	0.574	0.264	0.631
Na	mmol/L	0.607	0.094	0.259	0.421	0.792
Potassium	mmol/L	0.707	0.084	0.014	0.542 *	0.871 *
Cl	mmol/L	0.662	0.091	0.074	0.484	0.839

## Data Availability

The original contributions presented in this study are included in the article. Further inquiries can be directed to the corresponding author.
